# Reduced Genotoxicity of Gold Nanoparticles With Protein Corona in *Allium cepa*

**DOI:** 10.3389/fbioe.2022.849464

**Published:** 2022-04-05

**Authors:** Sagar S. Arya, James E. Rookes, David M. Cahill, Sangram K. Lenka

**Affiliations:** ^1^ The Energy and Resources Institute, TERI-Deakin Nanobiotechnology Centre, Gurugram, India; ^2^ School of Life and Environmental Sciences, Deakin University, Waurn Ponds Campus, Geelong, VIC, Australia

**Keywords:** vanillin capped gold nanoparticles, protein corona, plant, genotoxicity, chromosomal aberrations

## Abstract

Increased usage of gold nanoparticles (AuNPs) in biomedicine, biosensing, diagnostics and cosmetics has undoubtedly facilitated accidental and unintentional release of AuNPs into specific microenvironments. This is raising serious questions concerning adverse effects of AuNPs on off-target cells, tissues and/or organisms. Applications utilizing AuNPs will typically expose the nanoparticles to biological fluids such as cell serum and/or culture media, resulting in the formation of protein corona (PC) on the AuNPs. Evidence for PC altering the toxicological signatures of AuNPs is well studied in animal systems. In this report, we observed significant genotoxicity in *Allium cepa* root meristematic cells (an off-target bioindicator) treated with high concentrations (≥100 µg/ml) of green-synthesized vanillin capped gold nanoparticles (VAuNPs). In contrast, protein-coated VAuNPs (PC-VAuNPs) of similar concentrations had negligible genotoxic effects. This could be attributed to the change in physicochemical characteristics due to surface functionalization of proteins on VAuNPs and/or differential bioaccumulation of gold ions in root cells. High elemental gold accumulation was evident from µ-XRF mapping in VAuNPs-treated roots compared to treatment with PC-VAuNPs. These data infer that the toxicological signatures of AuNPs are influenced by the biological route that they follow to reach off-target organisms such as plants. Hence, the current findings highlight the genotoxic risk associated with AuNPs, which, due to the enhanced utility, are emerging as new pollutants. As conflicting observations on the toxicity of green-synthesized AuNPs are increasingly reported, we recommend that detailed studies are required to investigate the changes in the toxicological signatures of AuNPs, particularly before and after their interaction with biological media and systems.

## Graphical Abstract

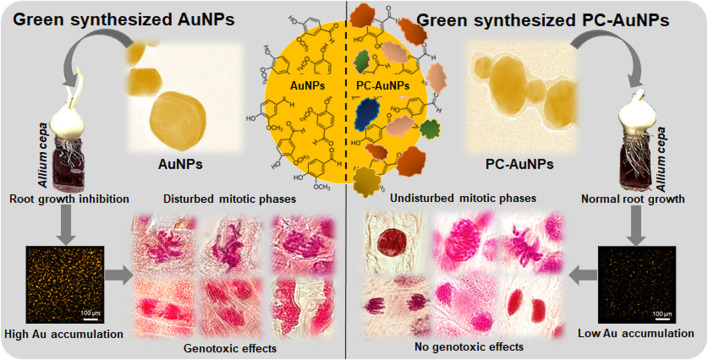

## Introduction

The global market of gold nanoparticles (AuNPs) is estimated to reach 6.33 billion USD by 2025 due to their increasing usage in biomedicine, biosensing, diagnostics and cosmetics (Grandviewresearch, 2019). AuNPs have gained wide application because of their chemical inertness, perceived low intrinsic cytotoxicity, customizable nanoscale morphology, prospects for surface functionalization and their plasmonic properties that are capable of inducing biological responses (Mosquera et al., 2020; Umapathi et al., 2020; Kumawat et al., 2022). However, this rapid increase in the use of AuNPs has enhanced the likelihood of their release into a specific microenvironment (Gomez et al., 2014) through their use such as that for functionalized/capped AuNPs that have been examined for cancer diagnosis/therapy (Sztandera et al., 2018), wound healing (Naraginti et al., 2016), drug delivery (Katsnelson et al., 2013; Kyriazi et al., 2018; Lopez-Chaves et al., 2018; Ansari et al., 2019), as cosmetic components (Cao et al., 2016), for chemical sensing (Qin et al., 2018) and antibacterial applications (Zong et al., 2017; Govindaraju et al., 2018). During such biological applications, AuNPs are exposed to biological fluids or cell culture media, which can result in the adsorption of proteins onto their surface, forming a so-called “protein corona” (PC) (Mosquera et al., 2020). This aggregated cloud of proteins has been attributed to altering or masking the functional signatures of green-synthesized AuNPs (Mosquera et al., 2020). With increasing reports of green-synthesized AuNPs being tested in animal cell cultures/models (Naraginti et al., 2016; Mukherjee et al., 2017; Ovais et al., 2018), and with promotion of AuNPs-supplemented cosmetics (Cao et al., 2016; Fytianos et al., 2020), it is important to evaluate and compare the toxicity of such engineered AuNPs with and without PC on off-target organisms. Previous toxicity studies have focused predominantly on animal cell cultures (Wheeler et al., 2021), suggesting that the relevance of PC formed on AuNPs, and the collective effect of such PC-AuNPs on off-target organisms is yet to be determined.

Since the 1980s, *A. cepa* has been extensively used to investigate the possible genotoxic effects of chemicals on off-target species (WHO, 1985). The United Nations Environment Programme and International Programme on Chemical Safety certify *A. cepa* as a standard bioindicator to analyze genotoxicity of environmental pollutants (Grant, 1982; WHO, 1985; Adair and Cobb, 1999). *Allium cepa* is also one of the best-suited models for *in vivo* cytogenotoxicity analysis of emerging environmental pollutants and is often used to predict and/or correlate the toxicity data with animal cell cultures/models (Maity et al., 2020). The prime advantages in using *A. cepa* as an *in vivo* model include easy monitoring of root growth in direct contact with the target chemical/material, since this species exhibits distinct phenotypic and genotypic responses to genotoxic materials (Bonciu et al., 2018). Further, the cytogenetic abnormalities of mitotic phases can be easily observed using optical microscopy as well as rapid and cost-effective assays that can be developed using root culture (Tedesco and Laughinghouse IV, 2012). Therefore, numerous studies have used *A. cepa*-based assays to test the genotoxicity of nanoparticles (Kumari et al., 2011; Pesnya, 2013; Saha and Gupta, 2017; Scherer et al., 2019; Liman et al., 2021). Collectively, these previous investigations indicate that spontaneous reactive oxygen species (ROS) generation occurs following cellular internalization of various types of nanoparticles, resulting in lipid peroxidation that leads to a decrease in the mitotic index and the triggering of chromosomal abnormalities. The system *A. cepa* was chosen based on the ease of availability and visualization of chromosomal aberrations in real time compared to other model plants, such as *Vicia* spp. and *Nicotiana tabacum*, respectively. Although *Nicotiana* spp. are used to assess nanotoxicity based on comet assay, root growth and biochemical parameters (Lovecká et al., 2021), are secondary observations.

While numerous studies have highlighted the genotoxic effects of silver and other metal nanoparticles on *A. cepa*, only a few reports describe the influence of AuNPs, which is surprising given the enormous research focus and potential industrial applications of AuNPs. There are some reports, which provide evidence for the genotoxic signatures of functionalized/capped AuNPs on *A. cepa* (Gopinath et al., 2013; Rajeshwari et al., 2016a; Rajeshwari et al., 2016b; Debnath et al., 2018). For instance, it was observed that the mitotic index of *A. cepa* meristematic cells treated with citrate-capped AuNPs was directly proportional to the concentration and inversely related to the size of AuNPs (Rajeshwari et al., 2016b). In the case of CTAB-capped Au nanorods, cyto-genotoxicity increased in a dose-dependent manner, whereas for PEG-capped gold nanorods the toxicity remained insignificant irrespective of the tested concentration range, which was 0.1–10 µg/ml (Rajeshwari et al., 2016a). Contrary to the above findings, AuNPs synthesized using *Sphaeranthus indicus* extract (1–10%) promoted mitotic cell division in *A. cepa* root tips depending on the concentration of extract used, with 10% of extract synthesized AuNPs showing the highest mitotic index (Balalakshmi et al., 2017). Similarly, studies performed on *Oryza sativa* (Ndeh et al., 2017), *Vigna radiata* (Das et al., 2017), and several other species (Venzhik et al., 2021), showed that AuNPs have the potential to enhance germination rate, growth and photosynthesis at higher concentrations, i.e., 10–1,000 µg/ml. Such contradictory observations towards AuNPs suggest that, AuNPs, irrespective of their origin and application, should be tested for the presence of off-target effects at high concentrations.

Presently, given the conflicting results in the literature describing the bioactivity and biocompatibility of engineered AuNPs in plants, there is no clear consensus towards the toxicological signature of AuNPs (Sani et al., 2021). Furthermore, none of the available data details how the PC can influence the toxicological signatures of AuNPs, specifically on an off-target plant model. In this regard, we investigated the genotoxic effects of green-synthesized vanillin capped gold nanoparticles (VAuNPs) (Arya et al., 2019), and protein-coated VAuNPs (PC-VAuNPs) on *A. cepa* (PC in the present study refers to the coating of proteins after treating VAuNPs with Fetal bovine serum). Vanillin (vanilla flavour) was used as a capping agent owing to its popularity as a flavouring molecule (Arya et al., 2022), as well as its widely reported bioactive potential (Arya et al., 2021a; Arya et al., 2021b). Moreover, we have earlier reported that vanillin and its gold nanoparticle formulations can be used as antibiotic resistance reversal agents. Organic capping agents like curcumin or citric acid are extensively explored for toxicity studies. Additionally, the outcome of the toxicity studies of vanillin capped AuNP can be correlated with curcumin capped AuNPs as well.

Due to the growing interest in green-synthesized AuNPs compared with those that are chemically synthesized, we used biomolecule capped AuNPs for our genotoxicity analysis. This report details the differences in the genotoxicity between bare and protein-coated AuNPs on the off-target model plant *A. cepa*. The outcomes provide important new information on the role of PC in defining the toxicological signatures of green-synthesized AuNPs.

## Materials and Methods

### Synthesis and Characterization of VAuNPs With and Without Protein Corona

VAuNPs were synthesized according to the previously described method (Arya et al., 2019). Briefly, 2 mM gold chloride (HAuCl_4_) was reduced with 1 mM vanillin (Sigma Aldrich, St. Louis, United States) for 3 h at room temperature. The synthesized VAuNPs were centrifuged (13,000 rpm, 20 min; Eppendorf™ Cooled Centrifuge 5424 R, Germany) and washed thrice with sterile deionized water.

To mimic the laboratory cell culture condition, the procedure to form PC around VAuNPs was performed as described earlier with slight modifications (Cheng et al., 2015; Mosquera et al., 2020). Briefly, the VAuNPs were incubated in fetal bovine serum [FBS, 10% in phosphate buffer saline (PBS)] at 37 ± 2°C for 24 h to form FBS-VAuNPs, i.e., PC-VAuNPs. After incubation, the VAuNPs suspension was centrifuged (13,000 rpm, 20 min; Eppendorf™ Cooled Centrifuge 5424 R, Germany) and the pellet formed was rinsed thrice with PBS to remove unbound proteins from VAuNPs. The synthesis of bare VAuNPs and PC-VAuNPs was confirmed by their characterization using UV-visible spectrophotometry (UV-2450, Spectrophotometer, Shimadzu, Kyoto, Japan), dynamic light scattering (Malvern Zetasizer Nano ZS, United States), transmission electron microscopy (TEM) (FEI Tecnai F20 TEM, Philips, Netherlands) and Fourier transform infrared spectrophotometry (FTIR) (Nicolet 6,700, Thermo Scientific, United States).

### *Allium cepa* Genotoxicity and Microscopic Analysis

The genotoxicity studies were performed in *A. cepa* with the synthesized VAuNPs as described earlier (Rajeshwari et al., 2016b). Equal-sized (25–30 mm) and healthy *A. cepa* bulbs, weighing 30–35 g obtained from a local vegetable market (Gurugram, India) were grown under dark conditions in an enclosed chamber. A temperature of 25 ± 2°C was maintained, and renewed water supply was provided for every 24 h. Roots of 2–3 cm were excised from the bulb and treated with various concentrations (0, 12.5, 25, 50, 100 and 200 µg/ml) of the VAuNPs. A treatment group exposed to 100 µM AgNO_3_ (16.98 µg/ml) was used as the positive control due to its proven genotoxicity and root growth inhibitory effects (Cvjetko et al., 2017). Ultrapure ion-free “MilliQ” water was used as a negative control. The aqueous colloidal solutions of VAuNPs and AgNO_3_ were prepared by resuspending and dissolving, respectively, in ultrapure ion-free MilliQ water. Three replicates were made for each concentration.

After 4 h of exposure, the root tips were removed and rinsed with sterile deionized water. Then, the root tips were immersed in 1 M HCl for 20 min and dipped in acetocarmine stain for 5 min (Rajeshwari et al., 2016b). The root tips were then rinsed to remove excess stain and approximately 1–2 mm sections were cut, using a scalpel, from each root tip. The sections were placed onto a glass slide and covered with a coverslip. The coverslips were gently pressed using light thumb pressure to prepare a uniformly thick section squash. The slides were examined under an optical microscope (Axiostar, Zeiss, Germany) and the genotoxic effect of VAuNPs and AgNO_3_ on the root tip cells were determined by scoring 10^3^ cells with three replicates per test concentration. The mitotic index, phase index, and the total number of cellular abnormalities observed in mitotic cells were calculated using the following formulas (Saha and Gupta, 2017).

$$Mitotic\;index\;\left(MI\right)\%=\left(\frac{TDC}{TC}\right)*100$$

$$Phase\;index\;\left(PI\right)\%=\left(\frac{TCP}{TDC}\right)*100$$

$$Chromosonal\;Aberrations\;\left(CA\right)\%=\left(\frac{TCA}{TC}\right)*100$$

Where, TDC was the total number of dividing cells, TC was the total number of cells counted, TCP the total number of cells in a particular mitotic phase and TCA the total number of cells that showed chromosomal aberrations.

Similarly, the protein coated VAuNPs (100 and 200 µg/ml) were analyzed for any genotoxic effects using the above protocol. Vanillin (1 mM) and Fetal Bovine Serum (10% FBS in sterile deionized water) were used as controls, based on their use in capping and coating, respectively.

### *Allium cepa* Root Growth Assay

A growth inhibition test was performed according to the protocol of Cvjetko et al. (2017). *A. cepa* bulbs were scraped and peeled to remove dried roots and outer shells, respectively to expose the root primordial apices. Bulbs were then placed on the top of flat bottom glass tubes (Borosil, India) (one bulb per tube) filled with different concentrations of VAuNPs (0, 12.5, 25, 50, 100, and 200 µg/ml), and kept in dark conditions at 25 ± 2°C for 96 h. The AgNO_3_ treatment of 100 µM (16.98 µg/ml) was used as a positive control. To determine the root length and EC_50_ value, the length of the five longest roots (30 roots from 3 bulbs) was measured from each control and treated onion bulb.

Similarly, the PC-VAuNPs (100 and 200 µg/ml) were analyzed for any root growth inhibition effects using the above-mentioned protocol. Vanillin (1 mM) and FBS (10% FBS in sterile deionized water) were used as controls.

### Estimation of Catalase Activity

Catalase (CAT) activity in the root tips cells was evaluated as previously described (Rajeshwari et al., 2015; Souza et al., 2020), with slight modification. Approximately 200 mg of root tips (e.g., a minimum of 10 roots) from each treatment of VAuNPs (0—200 µg/ml), AgNO_3_ (100 µM), vanillin (1 mM), FBS (10%) and PC-VAuNPs (100 and 200 µg/ml) were taken after 4 h of incubation and were homogenized manually in a chilled mortar and pestle at 4°C in 1.5 ml potassium phosphate buffer (100 mM, pH 7) containing 0.1 mM EDTA. The homogenate was centrifuged for 30 min at 10,000 rpm and 4°C (Eppendorf™ Cooled Centrifuge 5424 R, Germany), and then the enzyme-containing supernatant was separated and used to estimate CAT activity. The 3 ml reaction mixture consisted of 2.96 ml of potassium phosphate buffer (100 mM, pH 7) and 20 µl of enzyme extract. The reaction was started by adding 2.5 µl of 30% of hydrogen peroxide (H_2_O_2_). The activity was quantified based on the consumption of exogenous H_2_O_2_ by CAT, generating water and oxygen with a decrease in spectrophotometric absorbance at 280 nm.

### Histochemical Staining and Determination of Superoxide Radical

The production of a superoxide radical (O_2_^−^) was visually examined in *A. cepa* roots with histochemical staining using nitro blue tetrazolium to determine the level of oxidative stress. The presence of O_2_^−^ was detected as per the method of Mangalampalli et al. (2018). The roots were treated with VAuNPs (0—200 µg/ml), AgNO_3_ (100 µM), vanillin (1 mM), FBS (10%) and PC-VAuNPs (100 and 200 µg/ml) along with control for 4 h and then stained with 2 mM nitro blue tetrazolium (NBT; Sigma-Aldrich, United States) in 20 mM PBS (pH 6.8) for 10 min and subsequently washed with deionized water. Superoxide anions were observed as a deposit of dark blue insoluble formazan compounds (Soltys et al., 2011), and images were acquired with a digital camera (Olympus E-520 camera, OLYMPUS, Tokyo, Japan).

To estimate O_2_^−^ generation, the onion root tips were treated with VAuNPs (0—200 µg/ml), AgNO_3_ (100 µM), vanillin (1 mM), FBS (10%) and PC-VAuNPs (100 and 200 µg/ml) along with control for a sampling period of 4 h. After treatment, 100 mg of root tips were homogenized in 0.5 ml of freshly prepared 0.1% trichloro acetic acid using a precooled (4°C) mortar and pestle. The homogenate was incubated with 0.3 ml of reaction mixture containing Tris-HCl buffer (50 mM; pH 6.5), NBT (0.2 mM), nicotinamide adenine dinucleotide (0.2 mM), and sucrose (250 mM) for 30 min at 28 ± 2°C in the dark. The blue mono-formazan formed was measured at 530 nm and its concentration was calculated using an extinction coefficient of 12.8 L/(mol*cm), which provided an indirect measurement of the concentration of O_2_^−^ radical generated and was expressed as μmol NBT reduced in 0.1 g fresh weight of roots.

### Micro-XRF Mapping of Au in *Allium cepa* Roots

µ-XRF was performed to study the Au uptake in *A. cepa* roots as described by Tefera et al. (2020). Onion bulbs with roots that were 1 week old were transferred to VAuNPs and PC-VAuNPs colloidal solutions of 200 µg/ml for 24 h. After 24 h, the distal 1 cm of the roots were excised and gently rinsed with MilliQ water. The roots were then immediately frozen in liquid nitrogen and stored at–20°C prior to use for Au mapping.

Au mapping was performed using µ-XRF (XGT9000, Horiba Scientific, Kyoto, Japan). The electron voltage was maintained at 50 kV with a beam intensity of 1,000 µA. The fluorescence yield was detected using a silicon drift detector. The Au distribution was collected using a map size of 0.5 mm width and height. The elemental composition of *A. cepa* roots was determined for the meristematic and elongation zones.

### Statistical Analysis

All experiments were performed in triplicate. Means of pooled data with standard deviation were statistically compared to the control using One-way ANOVA followed by Duncan’s multiple range test. Data were analysed using a commercial statistics package (IBM SPSS Statistics 27.0) and means were considered statistically significant when *p* < 0.05.

## Results and Discussion

### Synthesis and Characterization of VAuNPs With and Without Protein Corona

**FIGURE 1:**
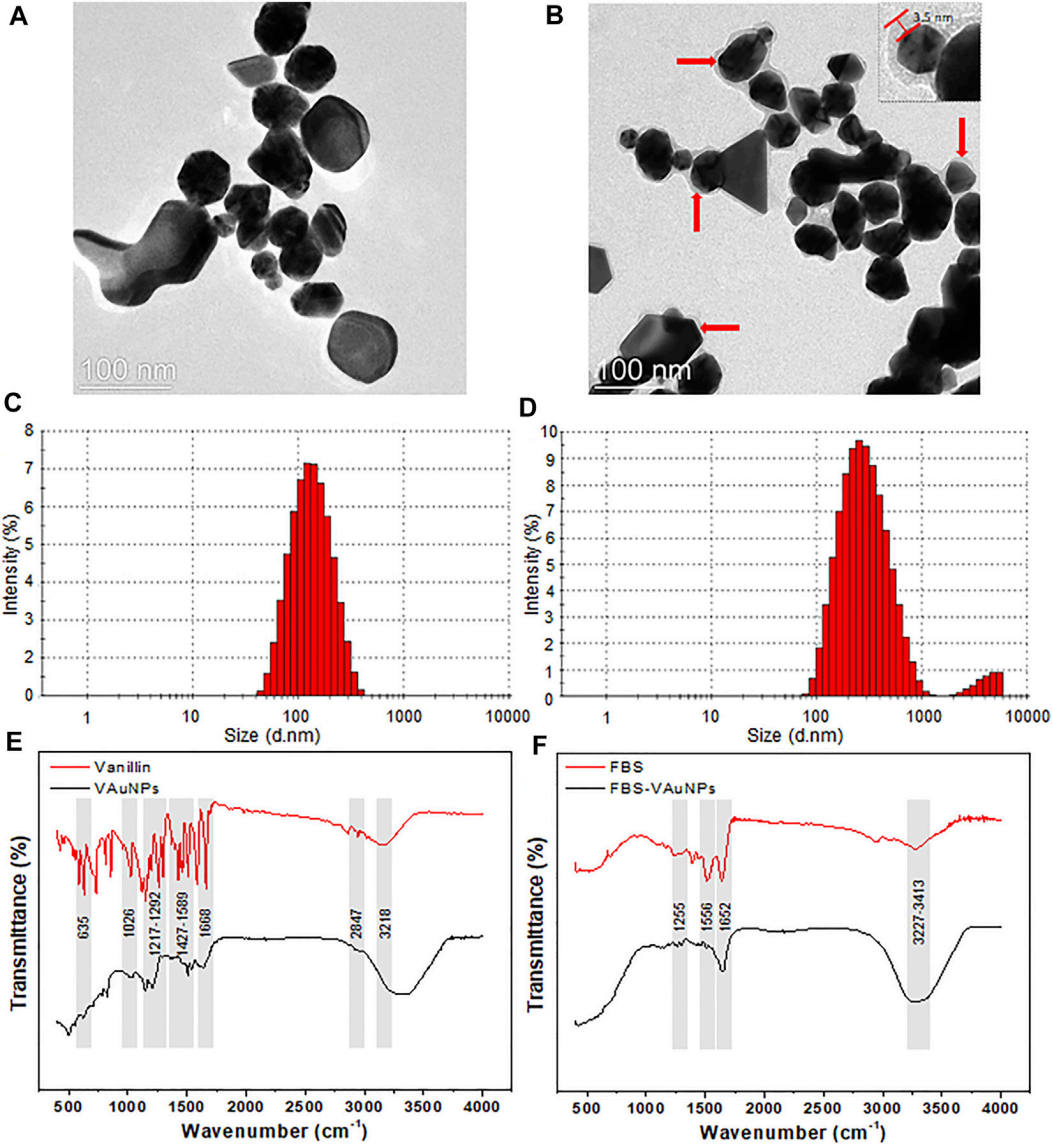
TEM, hydrodynamic size distribution and FTIR of bare VAuNPs **(A,C,E)**, and PC-VAuNPs **(B,D,F)**, respectively. Solid red arrows **(B)** show the build-up of protein coating with average thickness of 3.5 ± 1.73 nm, which appears as a grey shadow around dark VAuNPs.

The characterization of synthesized VAuNPs with and without PC revealed certain physiochemical differences. The UV-visible spectra originating from the optoelectronic surface plasmon resonance property confirmed the synthesis of VAuNPs, whereas the difference in the surface plasmon resonance of PC-VAuNPs and VAuNPs indicated aggregation of the PC-VAuNPs due to PC formation (Supplementary Figure S1). TEM analysis showed that the VAuNPs were mainly hexagonal, spherical, irregular and occasionally triangular in shape (Figure 1A). TEM images also revealed the formation of PC around VAuNPs when treated with 10% FBS, i.e., formation of PC-VauNPs (Figure 1B). The thickness of the PC around VAuNPs varied, but was on average 3.5 nm (Figure 1B). This observation is in accordance with the literature, which suggests that protein abundance of PC is variable and depends on the type and amount of proteins in the biological fluid (Liu et al., 2020), and in this case the presence of FBS. Dynamic light scattering showed an increase in the hydrodynamic size and zeta potential measurements of bare VAuNPs, compared to PC-VAuNPs (Figures 1C,D), and were recorded as 99.94 ± 3.41 and 226.14 ± 5.12, and -31.9 mV and +9.21 mV, respectively. Similarly to the increase in the nanoparticle size and surface charge reversal observed in our study, Li et al. (2021) reported that humic acid adsorption on AuNPs increases their effective size and reverses nanoparticle surface charge. Further, Boldeiu et al. (2019) reported that citrate- and honey-capped AuNPs suspended in culture media supplemented with 10% FBS resulted in the formation of PC around AuNPs, which promoted agglomeration and clustering of the AuNPs. Moreover, it has been observed that the kinetics of PC formation and the characteristics of PC are based on the particle size as well as their shape (Piella et al., 2017). For instance, the thickness of PC on AuNPs influences the hydrodynamic size of nanoparticles (Cheng et al., 2015). In addition, it is observed that smaller nanoparticles show more increase in their size after corona formation compared to larger ones. It is reported that, smaller particles are more stable and have higher surface area than the larger particles, which results in the faster dispersion of smaller particles due to increased Brownian motion and subsequent enhancement in the coating of available proteins (Piella et al., 2017). Thus, the size of PC on smaller VAuNPs is high compared to larger VAuNPs.

The FTIR spectra of VAuNPs revealed the presence of different functional groups of vanillin molecules capped on the surface of VAuNPs (Figure 1E). FTIR peaks observed at 635 cm^−1^ and 1,668 cm^−1^, 1,026 cm^−1^, 1,217–1,292 cm^−1^ and 3,218 cm^−1^, 1,427–1,589 cm^−1^, and 2,847 cm^−1^ correspond to stretching and bending vibrations of the aldehyde group (C=O), stretching vibrations of O-CH_3_, stretching (a broad band) and bending (a medium band) vibrations of the hydroxy group (-OH), stretching vibrations of C=C-C, and stretching vibrations of -C-H with weak band absorption, respectively (Aarabi et al., 2017; Fatoni et al., 2018; Arya et al., 2019). Similarly, the FTIR spectra of VAuNPs treated with FBS revealed the coating of proteins on the surface of VAuNPs (Figure 1F). The FTIR peaks observed at 1,255 cm^−-1^, 1,556 cm^−-1^, 1,652 cm^−-1^ and 3,227–3,414 cm^−1^ corresponds to C-O deformation vibrations in the carboxylic group, -NH bending vibrations of amide II and C-N stretching in -CO-NH- in proteins, stretching vibrations of C=O of amide I in proteins, stretching vibrations of -OH and -NH in hydroxy and amine groups, respectively (Song et al., 2016). The FTIR of FBS treated VAuNPs suggests that several functional groups of vanillin present on VAuNPs are masked by proteins from FBS to form a PC on VAuNPs (Figures 1E,F). The stretching vibrations arising from -OH group on VAuNPs (Figure 1E) are severely contracted, as well as the intensity of the absorption bands in PC-VAuNPs (Figure 1F). Increase in the FTIR peak intensity in the range 3,227–3,414 cm^−1^ of PC-VAuNPs (Figure 1F) compared to bare VAuNPs (Figure 1E) suggests the successful coating of hydroxy group-containing amino acids such as serine, threonine and tyrosine. Another plausible explanation to the observed reduction of vibrational modes is that protein conjugation may have resulted in the exchange of vanillin from the surface of VAuNPs.

### *Allium cepa* Genotoxicity Assessment for VAuNPs With and Without Protein Corona

AuNPs are certainly among the most preferred metal nanoparticles for biomedical and biosensing applications owing to their tuneable plasmonic properties and chemically inert nature. However, such increase in their use poses considerable risk of intentional/unintentional release and accumulation of AuNPs into the target environment, often presenting a threat towards off-target organisms such as plants. For instance, reports of an animal model (male wistar rats) administered with AuNPs suggested that AuNPs were excreted through faeces and urine (Katsnelson et al., 2013; Lopez-Chaves et al., 2018; Ansari et al., 2019). Additionally, the AuNPs used in cosmetics (Cao et al., 2016), which interact with biological fluids secreted by the dermal layer are washed-off and released into sewage water. These studies clearly indicate that AuNPs would eventually enter the environment if they are used as a part of nanomedicines/cosmetics. However, AuNPs from such sources will be coated with biomolecular corona which may alter the functional signatures of AuNPs. Similarly, inappropriate handling of nanoparticles can lead to their spread in the form of aerosols (Gomez et al., 2014). Therefore, it is important to assess and compare the toxicological signatures of such biomedically relevant AuNPs with and without biomolecular corona (here PC) on off-target organisms. To address this, the effect of some chemically and green synthesized AuNPs have been tested in root cells in the off-target model organism *A. cepa*, although only at relatively low concentrations (0.1–10 µg/ml) (Rajeshwari et al., 2016a; Rajeshwari et al., 2016b; Debnath et al., 2018). These studies suggested that AuNPs induce genotoxicity by generating oxidative stress in the root cells in a dose dependent manner. Interestingly, several studies have shown that at much higher concentrations (400–1,000 µg/ml), AuNPs can promote growth and germination in rice (*Oryza sativa*) (Ndeh et al., 2017), Mung Bean (*Vigna radiata*) (Das et al., 2017), and other plant species (Venzhik et al., 2021). However, there is no report that evaluates and compares, on plants, the effect of AuNPs with and without PC.

**FIGURE 2:**
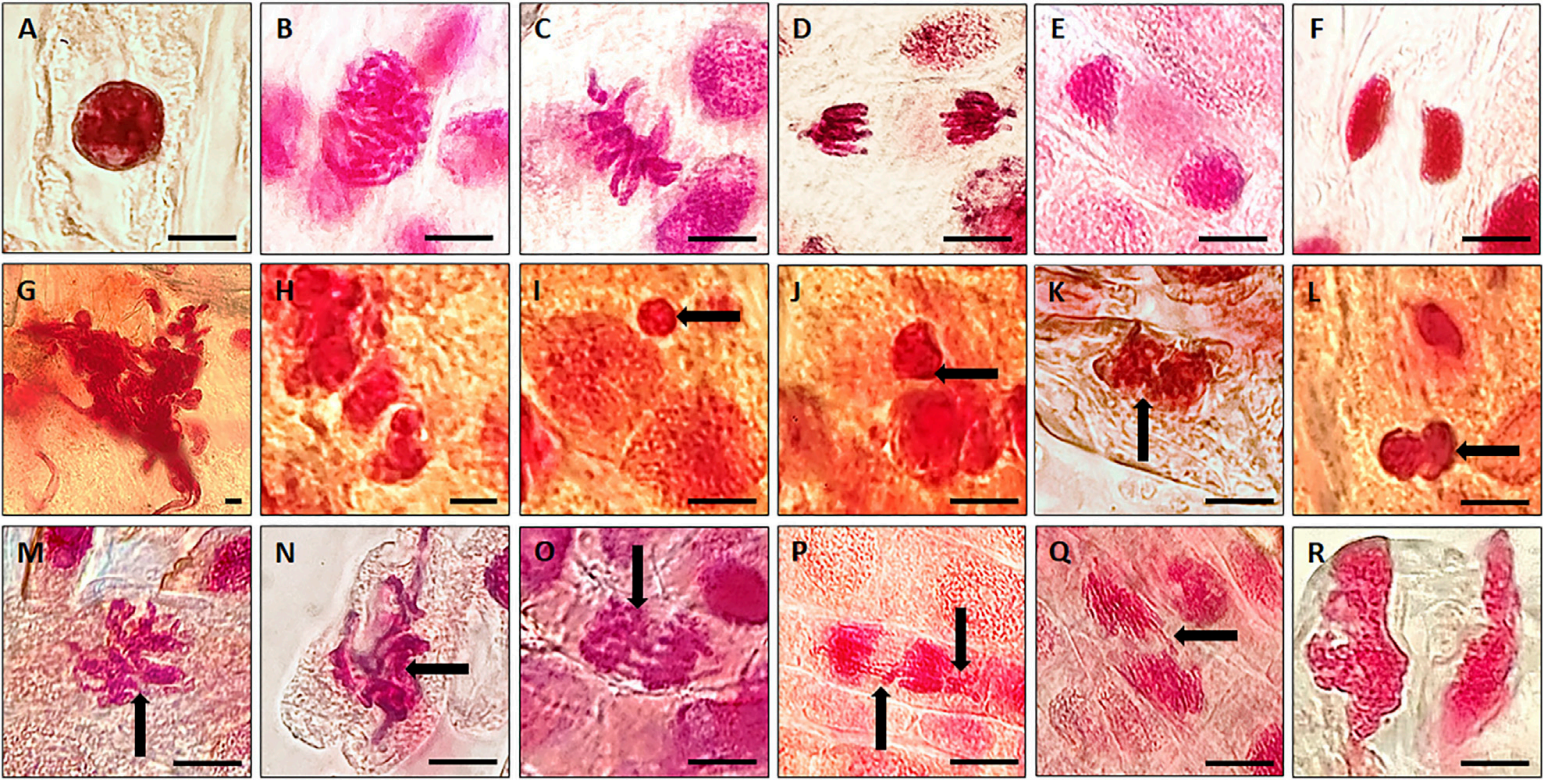
Representative photographs of individual phases of mitosis observed in untreated (control) *A. cepa* root tip cells; **(A)** Interphase; **(B)** Prophase; **(C)** Metaphase; **(D)** Anaphase; **(E)** Telophase; **(F)** Cytokinesis. Representative images of chromosomal and nuclear anomalies more frequently observed following either exposure to higher concentrations of VAuNPs (100 and 200 µg/ml) or 100 µM AgNO_3_ in dividing cells of *A. cepa* roots; **(G)** nuclear clumping and lesions, **(H,I)** micronuclei, (Black arrow shows the feature) **(J–L)** binucleate, **(M)** sticky chromosomes at metaphase, **(N,O)** disturbed metaphase, **(P,Q)** anaphase bridge and laggard chromosome, **(R)** disturbed telophase. (Scale 10 µm). Here, both VAuNPs and AgNO_3_ treatments produced similar chromosomal/nuclear anomalies. Representative images of *n* ≥ 10^3^ cells for each treatment.

Therefore, in the present study we considered and tested a range of concentrations, i.e., 12.5–200 µg/ml of VAuNPs [assuming that vanillin capping would increase the biocompatibility of AuNPs, as mentioned for phytomolecules by Cao et al. (2017)] to assess their genotoxicity in *A. cepa* root tip cells. Based on the outcome of VAuNPs treatments, we tested PC-VAuNPs at 100 and 200 µg/ml concentrations. Table 1 shows the percentage of mitotic index, phase index and chromosomal aberrations observed when *A. cepa* root tips were incubated with different concentrations of VAuNPs, where AgNO_3_ and ultrapure ion free MilliQ water is used as positive and negative controls, respectively. The MI values of positive and negative control samples were 27.13 ± 1.93 and 45.70 ± 1.67, respectively. In comparison to the negative control, a significant reduction in the frequency of different dividing stages was observed at higher concentrations of treatment groups such as 100 µg/ml and 200 µg/ml. Similarly, significant increases in chromosomal and nuclear abnormalities were observed at these concentrations, which corresponded with that for the positive control. A concentration-dependent decrease in the MI values was recorded with an increasing concentration of VAuNPs (Table 1). The MI value (25.76 ± 1.06) of 200 µg/ml VAuNPs, which is below that for the AgNO_3_ treatment (27.13 ± 1.93) clearly highlights the genotoxic effects of VAuNPs at higher concentrations. Mitotic cells of the untreated negative control cells showed the expected phases of cell division such as prophase, metaphase, anaphase and telophase (Figure 2).

**TABLE 1:** Phase index (PI), mitotic index (MI) and chromosomal aberrations (CA) of *A. cepa* root meristematic cells treated with VAuNPs (0—200 µg/ml), AgNO_3_ (100 µM), Vanillin (1 mM), FBS (10%) and PC-VAuNPs (100 and 200 µg/ml).

Treatment	Conc	PI (% ± SD)				MI (% ± SD)	CA (% ± SD)
		Prophase	Metaphase	Anaphase	Telophase		
VAuNPs (µg/ml)	0 (water control)	96.71 ± 2.64^ab^	1.89 ± 0.05^f^	0.72 ± 0.12^def^	0.43 ± 0.19^cd^	45.70 ± 1.67^a^	0.06 ± 0.04^e^
	12.5	95.49 ± 0.84^c^	2.51 ± 0.22^cde^	0.91 ± 0.18^def^	0.68 ± 0.18^bcd^	43.70 ± 0.71^ab^	0.66 ± 0.12^de^
	25	94.98 ± 1.82^d^	2.82 ± 0.07^cde^	0.88 ± 0.12^def^	0.64 ± 0.10^cd^	41.23 ± 0.82^c^	0.83 ± 0.16^de^
	50	92.16 ± 1.83^e^	3.25 ± 0.16^bc^	1.33 ± 0.30^cd^	1.25 ± 0.39^b^	40.00 ± 0.49^c^	1.06 ± 0.18^d^
	100	89.86 ± 3.34^f^	4.48 ± 0.37^b^	2.13 ± 0.16^bc^	2.88 ± 0.31^a^	31.23 ± 0.78^d^	2.83 ± 0.33^c^
	200	86.15 ± 0.45^h^	6.20 ± 0.28^a^	3.10 ± 0.50^ab^	3.88 ± 0.28^a^	25.76 ± 1.06^e^	4.53 ± 0.49^b^
AgNO_3_ (µM)	100	89.92 ± 7.50^g^	3.56 ± 0.51^bc^	3.31 ± 0.20^a^	1.35 ± 0.37^bc^	27.13 ± 1.93^e^	15.90 ± 1.12^a^
Vanillin (mM)	1	96.55 ± 2.25^bc^	1.98 ± 0.31^f^	0.68 ± 0.19^ef^	0.30 ± 0.10^d^	43.60 ± 0.66^ab^	0.13 ± 0.04^e^
FBS (%)	10	96.91 ± 1.32^a^	2.27 ± 0.27^def^	0.44 ± 0.17^f^	0.22 ± 0.18^d^	45.36 ± 0.77^a^	0.10 ± 0.08^e^
PC-VAuNPs (µg/ml)	100	95.27 ± 2.30^c^	2.55 ± 0.33^cde^	0.97 ± 0.19^de^	1.12 ± 0.18^b^	44.43 ± 0.89^a^	0.53 ± 0.12^de^
	200	93.34 ± 3.08^d^	2.88 ± 0.17^bcd^	1.28 ± 0.31^cd^	1.92 ± 0.17^a^	41.45 ± 0.38^bc^	0.83 ± 0.16^de^

Data represented as mean ± SD, alphabets indicate significant difference from the control according to Duncan’s multiple range test.

With the reduction in MI values, the frequency of chromosomal and nuclear aberrations increased in all the treatment groups. It is reported by Saha and Gupta (2017) that similar to mutagenic agents, toxic chemicals or nanoparticles can decrease the mitotic activity of the treated cells by inhibiting the cells from entering prophase and restricting them to interphase or G_2_ phase. The reduction in MI is also attributed to blockage at the G1 stage that leads to the suppression of DNA synthesis. In addition, the pole shift by the depolymerization of spindle fibers led to the occurrence of different chromosomal aberrations in metaphase and anaphase (Rajeshwari et al., 2016b). The disturbance in spindle fibers followed by improper chromosome folding attributed to the formation of sticky chromosomes and chromosomal bridges, which cause the fragmentation of the chromosomes, leading to chromosomal anomalies. Thus the occurrence of chromosomal aberrations of root meristem cells indicates genotoxicity of the tested compounds (Smaka-Kincl et al., 1996). Chromosomal and nuclear abnormalities observed in the various treatments included nuclear clumping and lesions (Figure 2G), micronuclei (Figures 2H,I), binucleate cells (Figures 2J–L), sticky chromosomes at metaphase (Figure 2M), disturbed metaphase (Figures 2N,O), anaphase bridge and laggard chromosome (Figures 2P,Q), and disturbed telophase (Figure 2R).

A study performed with *Nicotiana xanthi* suggested that AuNPs (3.5 and 18 nm) were internalized by this species in a size-dependent manner and caused phytotoxicity after entering plant cells and subcellular organelles (Sabo-Attwood et al., 2012). Thus, AuNPs have the potential to induce changes in stress response genes, which can impede major cellular processes even following short-term AuNP exposure (Rajeshwari et al., 2016b). Debnath *et al.* (2018) reported that green-synthesized AuNPs between 50 and 100 nm reduced the MI in *A. cepa* cells in a concentration-dependent manner. Contrastingly, Gopinath et al. (2013) found that AuNPs prepared using a *Terminalia arjuna* leaf extract showed an increase in the MI and PI in a concentration-dependent manner (10–1,000 µM). However, in both these studies, any potential genotoxic effect of the plant extract alone was not evaluated, therefore making it difficult to determine whether the changes in the genotoxic signatures were due to AuNPs, phytochemicals or a combination of both. In a related study, Rajeshwari et al. (2016b) studied the effect of both AuNPs and citrate, which was used to synthesize the AuNPs. They observed that the capping agent, citrate, had no impact on the MI and PI, suggesting that genotoxicity was a function of the AuNPs. Using a similar approach, we observed that the capping agent, vanillin, and FBS, did not exhibit any genotoxicity effects when tested individually (Table 1).

Apart from AuNPs, some studies have been carried out with other metallic nanoparticles. For example, zinc oxide nanoparticles (ZnONPs) used at similar concentrations and size as used here induced chromosomal aberrations in *A. cepa* root tip cells (Kumari et al., 2011). Analysis of *A. cepa* root cells in that study by SEM and TEM showed ZnONP-like deposits inside the cell-matrix of 100 nm in size and also confirmed the internalization of ZnONPs and their agglomeration. It was also reported in this study that post-internalization, the nanoparticles were transported through plasmodesmata, during which the nanoparticles may aggregate, resulting in an increase in their size. For MgONPs of 50 nm in size, a concentration-dependent decrease in MI values was observed when treated with 12.5 µg/ml to 100 µg/ml MgONPs (Mangalampalli et al., 2018). Similarly, many other nanoparticles such as AgNPs (<60 nm) (Saha and Gupta, 2017), Al_2_O_3_NPs (<50 nm) (Rajeshwari et al., 2015), Cr_2_O_3_NPs (hydrodynamic size: 0—500 nm) (Kumar et al., 2015), TiO_2_NPs (<25 nm) (Fadoju et al., 2020), and BONPs (Bismuth III oxide nanoparticles) (90—210 nm) (Liman, 2013) were reported to be genotoxic at lower or similar concentrations.

To evaluate the genotoxic effects, the *A. cepa* assay was examined using higher concentrations of PC-coated VAuNPs (PC-VAuNPs) (100 µg/ml and 200 µg/ml). The PC-VAuNPs when used at these higher concentrations did not show any genotoxicity in cells, which was also confirmed from the deduced MI and PI values of the treated root meristem cells (Table 1). FBS (10%) and Vanillin (1 mM) treatments were used as controls for PC-VAuNPs. The MI of root meristems for 100 µg/ml and 200 µg/ml PC-VAuNPs treatments were 44.43 ± 0.89 and 41.56 ± 0.38, respectively, which were significantly less than that for the bare VAuNPs treatments at the higher concentrations (Table 1). Similarly, a significant reduction was observed in the number and frequency of chromosomal aberrations in PC-VAuNPs treatments at both 100 µg/ml and 200 µg/ml concentrations compared to VAuNPs (Table 1). The possible explanation for the differences in the genotoxicity between bare VAuNPs and PC-VAuNPs is related to their physicochemical properties, most importantly the hydrodynamic size and zeta potential. It has been reported that increased hydrodynamic size and agglomeration occurs when the zeta potential of nanoparticles skews towards neutral, which retards the electrostatic repulsion between the nanoparticles, thereby resulting in enhanced aggregation, and reduced bioavailability and toxicity. In general, low zeta potential values between–20 mV and +20 mV favours aggregation of nanoparticles (Ye et al., 2018). Similar to this observation, addition of the positively charged amino acid, Lysine, to suspended AuNPs (−34.8 mV) accelerated the agglomeration of AuNPs and significantly reduced their zeta potential to 7.2 mV (Ye et al., 2018). Also, it has been reported that translocation efficiency from root to shoot of AuNPs that have a negative zeta potential is greater than AuNPs with positive or neutral zeta potential values (Zhu et al., 2012). This signifies that AuNPs with negative zeta potentials are more likely to be rapidly internalized by the root and translocated to the shoot, whereas AuNPs with either a positive or neutral zeta potential adhere to and remain at the root surface.

A recent report (Janani et al., 2021), has detailed the influence of PC on the toxicity of ZnONPs in *A. cepa*. The ZnONPs with PC exhibited less cytotoxicity, chromosomal aberrations and micronuclei formation compared to ZnONPs without PC. Further, though not in plants, Li et al. (2021) demonstrated that humic acid adsorption onto AuNPs increases their size and hydrophobicity and reverses the surface charge, which sterically hinders the binding of AuNPs to bacterial cell membrane. Thus, the toxicity of nanoparticles is mitigated by a decrease in the chance of AuNPs association with bacteria, and AuNPs-induced membrane damage. Such findings provide compelling evidences for how modified surfaces change their toxicological signatures.

Nevertheless, to date, most studies targeted to evaluate the effect of PC on the toxicity of metal nanoparticles (particularly AuNPs) have been performed on animal cells or systems. Moreover, they were targeted to compare the mechanism of internalization of bare and PC-coated nanoparticles as well as to evaluate their toxicity. Collectively, the animal cell studies have concluded that the surface modification of nanoparticles by protein adsorption leads to reduced toxicity (Falahati et al., 2019). Further, the toxic effects are often attributed to the interaction of nanoparticles with the cell membrane, whereas Cheng et al. (2015) suggest that the modification of the nanoparticles with a corona protein coat eliminates such direct contact and decreases their toxicity. Additionally, the PC influences cellular uptake of AuNPs by phagocytic and nonphagocytic cells in a size-dependent manner (Cheng et al., 2015). It was also reported that the association of anionic nanoparticles with serum proteins reduces the likelihood of their interaction with animal cells (Charbgoo et al., 2018). Clathrin- and Cavelin-mediated endocytosis are reported to be one of the major mechanisms for internalization of PC coated AuNPs of 20–50 nm in size (Charbgoo et al., 2018). Contrary to animal cells, a clathrin-independent pathway is likely involved in AuNPs uptake in plant cells, and has been reported for cysteine functionalized AuNPs of –31.9 mV zeta potential (Li et al., 2016). In that study, the uptake of AuNPs in rice and tomato was blocked by specific inhibitors such as ikarugamycin and wortmannin (inhibitors of clathrin-mediated and clathrin-independent endocytosis, respectively). These results with inhibitors suggested that the uptake of AuNPs involved a combination of both clathrin-dependent and -independent mechanisms. In this regard, it would be of interest to investigate and compare the uptake mechanisms into plant cells that AuNPs with and without PC follow.

### *Allium cepa* Root Growth Assay for VAuNPs With and Without Protein Corona

**FIGURE 3:**
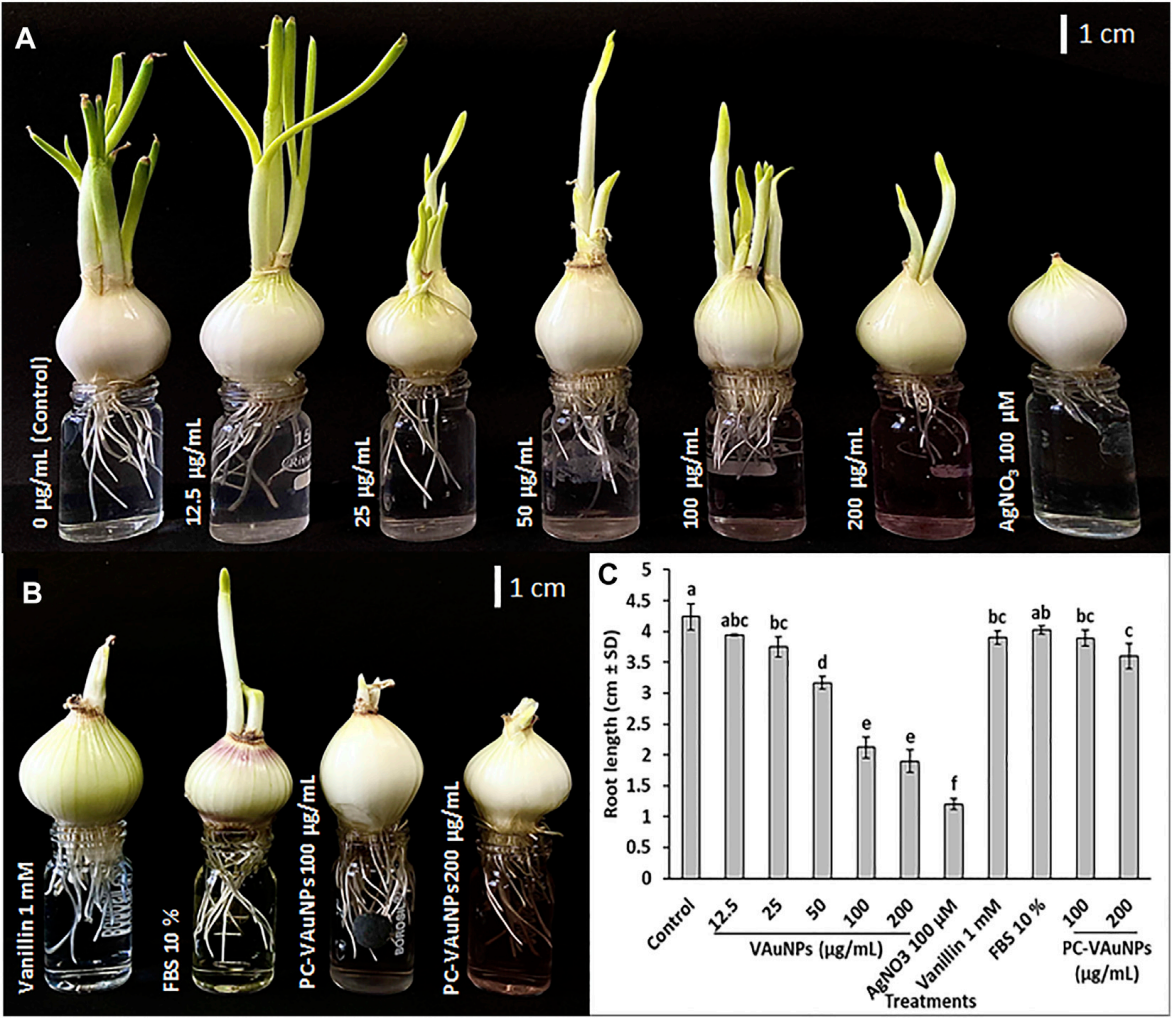
Allium cepa root growth assay **(A)** *A. cepa* root growth inhibition observed at different VAuNPs concentrations (12.5—200 µg/ml) after 96 h, AgNO_3_ was used as positive control; **(B)** *A. cepa* root growth inhibition assay of protein coated VAuNps (PC-VAuNPs, 100 and 200 µg/ml), after 96 h; **(C)** Effect of VAuNPs (0—200 µg/ml), AgNO_3_ (100 µM), Vanillin (1 mM), FBS (10%) and PC-VAuNPs (100—200 µg/ml) on root growth of *A. cepa*. Data represented as mean ± SD, bars with different letters are significantly different from the control according to Duncan’s multiple range test.

The *A. cepa* root growth assay revealed that VAuNPs significantly inhibited root growth in a concentration-dependent-manner (Figures 3A–C), which is consistent with the previous findings of a decrease in the MI values with increasing concentration of VAuNPs. The effective concentration (EC_50_) value, which retards 50% of root growth compared to the negative control, was found to be 100 µg/ml. The development and growth of *A. cepa* roots in 100 µg/ml and 200 µg/ml treatments were severely inhibited, with average root length of <2 cm after 96 h following exposure. It was observed that AuNPs of size 10, 14 and 18 nm inhibited the elongation of the primary root and lateral root formation in *Arabidopsis thaliana* at a concentration of 100 µg/ml (Siegel et al., 2018). Similarly, root growth inhibition was observed in Hordeum vulgare plants when exposed to citrate-stabilized AuNPs, at a concentration range of 3—10 µg/ml, whereas a lower concentration of AuNPs (1 µg/ml) promoted root growth (Charbgoo et al., 2018). Similar to VAuNPs, Deng et al. (2016) reported that CuONPs of 40 nm inhibited the growth of *A. cepa* roots in a concentration-dependent manner.

Unlike bare VAuNPs, high concentrations of PC-VAuNPs showed no negative impact on the growth of *A. cepa* roots (Figures 3A–C). The reason for the reduced genotoxicity of PC-VAuNPs is most likely related to their increased hydrodynamic size and their agglomeration due to reduced zeta potential. Similarly, Ding et al. (2019) reported that humic acid adsorption onto AgNPs significantly reduced the damage caused to the plant’s (*Lemna minor*) morphology and ultrastructure in comparison to bare AgNPs. Further, Ekvall et al. (2021) demonstrated that preexposure of Cobalt nanoparticles to biomolecules, which are always present in natural ecosystems reduced their toxic potency to *Daphnia magna*.

### Determination of Catalase Activity in *Allium cepa* Root Tips

It has been reported that the generation of ROS such as superoxide (O_2_^−^), hydroxyl (OH^−^), and hydrogen peroxide (H_2_O_2_) may be the primary cause of root growth inhibition in *A. cepa* when exposed to AuNPs (Rajeshwari et al., 2016b). The analyses performed in the previous study revealed that oxidative stress was an important mechanism linked to induction of cellular toxicity. Toxicity was likely promoted by factors such as oxidative groups functionalized on the surface of the nanoparticles followed by active redox cycling and interaction of nanoparticles with cellular components.

**FIGURE 4:**
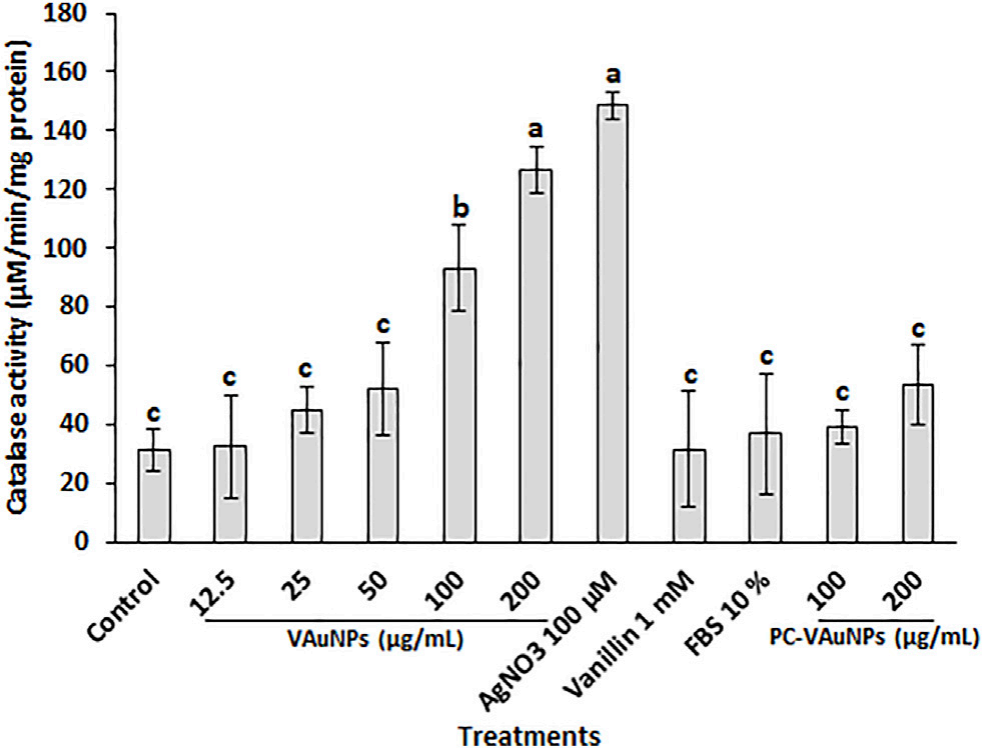
Catalase activity in *A. cepa* root tips following interaction with VAuNPs (0—200 µg/ml), AgNO_3_ (100 µM), vanillin (1 mM), FBS (10%) and PC-VAuNPs (100 and 200 µg/ml) for 4 h. Data represented as mean ± SD, bars with different letters are significantly different from the control according to Duncan’s multiple range test.

CAT is one of the major enzymatic ROS scavengers, which functions as efficient machinery for the detoxification of ROS such as H_2_O_2_. Gechev et al. (2006), reported that catalase was active only at relatively high H_2_O_2_ concentrations in plant systems. The CAT assay used here confirmed that VAuNPs generated oxidative stress in the *A. cepa* root tip cells in a concentration-dependent manner (Figure 4). In comparison, treatment of root tips with PC-VAuNPs generated significantly lower amounts of CAT activity compared to the bare VAuNPs at the same concentrations (100 and 200 µg/ml) and the positive control (100 µM AgNO_3_). More than a two-fold decrease in the catalase activity was observed in 200 µg/ml PC-VAuNPs treatment compared to bare VAuNPs of similar concentration.

It is reported that post internalization into plant cells, AuNPs are transported from 1 cell to another *via* plasmodesmata along with accumulation occurring inside the plant cell wall (Zhang et al., 2022). However, as evidenced from the characterization studies, the PC formed on VAuNPs and the resultant increased hydrodynamic size is likely to be responsible for the reduced toxicity and rate of internalization, respectively. Thus, the outcome of the present study corresponds to the previous findings of reduced genotoxicity and increased root growth in PC-VAuNPs compared to bare VAuNPs.

### Histochemical Staining and Determination of Superoxide Radical

**FIGURE 5:**
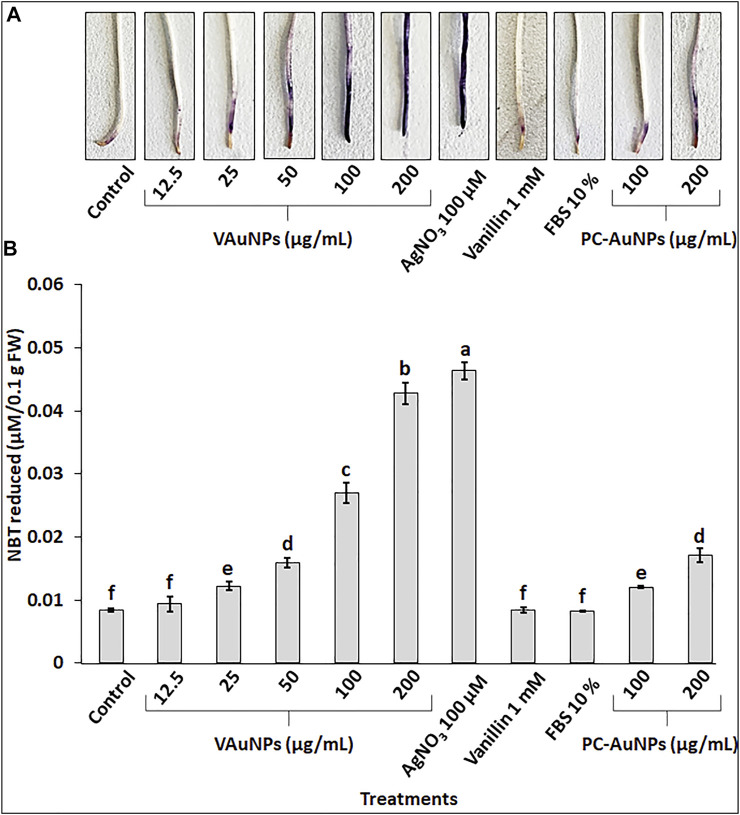
Visualization **(A)** and quantitation **(B)** of super oxide radical by NBT staining in roots of *A. cepa* treated with VAuNPs (0—200 µg/ml), AgNO_3_ (100 µM), vanillin (1 mM), FBS (10%) and PC-VAuNPs (100 and 200 µg/ml) along with control. Data represented as mean ± SD, alphabets indicate significant difference from the control according to Duncan’s multiple range test.

The production of O_2_^−^ was examined in *A. cepa* roots with histochemical staining using NBT for qualitative determination of the level of oxidative stress when roots were treated with VAuNPs (0—200 µg/ml), AgNO_3_ (100 µM), vanillin (1 mM), FBS (10%) and PC-VAuNPs (100 and 200 µg/ml) along with the water control (Figure 5A). The intensity of dark blue insoluble formazan (blue) staining increased based on O_2_^−^ production in a concentration-dependent manner in the VAuNPs treatments. Whereas, the level of O_2_^−^ produced in the PC-VAuNPs (100 and 200 µg/ml) treatments was much lower compared with similar concentrations of VAuNPs, and 100 µM AgNO_3_. A similar concentration-dependent effect on O_2_^−^ generation was observed in *A. cepa* roots treated with magnesium oxide nanoparticles (0—100 µg/ml) (Mangalampalli et al., 2018).

Quantitative analysis of O_2_^−^ in *A. cepa* roots confirmed the generation of oxidative stress in all the treatments (Figure 5B). The O_2_^−^ generation in VAuNPs treatments was enhanced in a concentration dependent manner. Further, as visualized in histochemical staining (Figure 5A), a significant difference was observed in the level of O_2_^−^ in VAuNPs and PC-VAuNPs treatments at the higher concentrations (100 and 200 µg/ml).

### Micro-XRF Mapping of Gold in *Allium cepa* Roots

**FIGURE 6:**
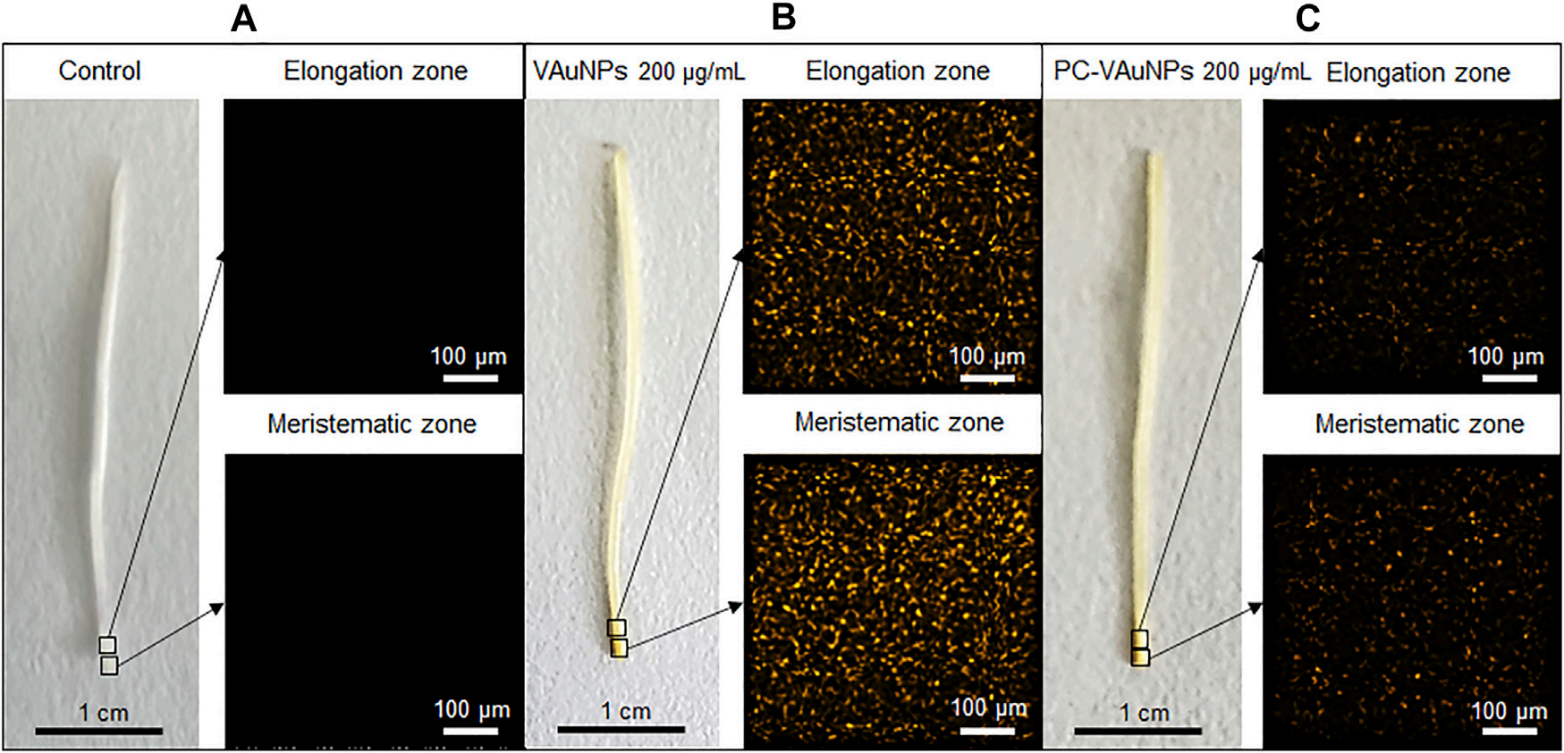
Micro-XRF mapping of elemental gold in elongation and meristematic zone of *Allium cepa* roots treated with; **(A)** Water control, **(B)** VAuNPs (200 µg/ml) and **(C)** PC-VAuNPs (200 µg/ml).

The bio-uptake of gold ions along a longitudinal section of *A. cepa* roots treated with VAuNPs and PC-AuNPs 200 µg/ml was determined using µ-XRF. Pixel intensity (Figure 6) indicated the level of gold fluorescence in treated and control roots. As compared to the water control treatment (Figure 6A), µ-XRF maps obtained from the fluorescence intensity of elements clearly indicated gold ion uptake within 24 h in VAuNPs and PC-VAuNPs (Figures 6B,C) treated samples. However, the difference in the intensities between VAuNPs and PC-VAuNPs is indicative of low-toxicity of PC-VAuNPs even when used at the highest concentration tested of 200 µg/ml. Supplementary Table S1 (provided at the bottom of the MS) details the presence of predominant elements in treated and control roots. Notably, a high intensity of elemental gold was obtained in the VAuNPs treatment which may be due to the uptake of both VAuNPs and ionic gold. For instance, it has been reported, that metallic nanoparticles may release a substantial fraction of metal ions in aqueous solution, and for such nanoparticles impacts on the test organism may be partly due to metal-ion-related toxicity (He et al., 2015). A similar study on *A. thaliana* roots exposed to gold ionic stress (200 µM) increased the H_2_O_2_ level in root tip tissues (Li et al., 2018), which may indicate, in the present study, that enhanced catalase activity is related to cellular toxicity of gold ions in the higher concentration of VAuNPs treatments. Taylor et al. (2014) reported that gold is taken up by the plants (*Arabidopsis thaliana* L.) predominantly in the ionic form when compared with AuNPs, and that plants respond to gold by upregulating the stress related genes and downregulating specific metal transporters to reduce the gold uptake. In the present study, PC formation on VAuNPs may have reduced the rate of gold ionization at the interface of VAuNPs and water, and an increase in hydrodynamic size likely hampered the internalization of the nanoparticles, which eliminated the toxicity arising from both ionic gold and VAuNPs. Similar to the present study, Ding et al. (2019) demonstrated that humic acid adsorption inhibited the absorption and accumulation of Ag in *Lemna minor* roots and leaves, which also reduced the toxicity associated with AgNPs.

## Conclusion and Prospects

The genotoxic effects of VAuNPs and PC-VAuNPs were assessed in the off-target model organism *A. cepa*. A dose-dependent decrease in MI values and an increase in chromosomal aberrations were observed in root meristems when treated with VAuNPs. Different types of chromosomal and nuclear anomalies observed here suggest that VAuNPs had adverse effects on root tip cells. The genotoxic effect was quite profound at higher concentrations of VAuNPs, whereas, treatment with PC-VAuNPs demonstrated a remarkable reduction in the frequency of chromosomal aberrations, and they had no impact on the MI and PI compared to bare VAuNPs. A higher accumulation of gold in roots, recorded by µ-XRF mapping, in VAuNPs treated roots compared to that found for PC-VAuNPs treated roots may underlie the genotoxicity found in the root tip cells. The outcomes of our studies show that the formation of PC can significantly reduce the genotoxicity of VAuNPs. As there are conflicting reports on the status of green-synthesized AuNPs, we recommend that more studies are required that focus on characterization of the nanoparticles and changes in their physicochemical properties before and after their exposure to biological media and systems. Understanding of the potential toxicity and mutagenicity of AuNPs on off-target organisms is required due to the current focus of research on biomedical/antibacterial applications and their use in cosmeceutical formulations. Therefore, attention needs to be drawn to the development of regulatory tools that may limit, during their utilization, AuNP release into the environment. Considering our current findings and those of previous studies, it is clear that AuNPs may impose cyto-genotoxicity irrespective of their method of synthesis. The research presented here supports a growing scientific consensus that our current knowledge of engineered AuNPs is inadequate, especially if we consider their effects on off-target organisms. However, further studies are recommended to elucidate the internalization mechanisms as well as the rate of internalization of PC and bare VAuNPs. Also, besides the present dose-dependent study, time- and shape-dependent toxicological investigations of gold salt, VAuNPs and PC-VAuNPs, as well as analysis of their interaction with other biomolecules is warranted.

## Data Availability

The original contributions presented in the study are included in the article/Supplementary Material, further inquiries can be directed to the corresponding author.

## References

[B1] Aarabi A. Mizani M. Honarvar M. (2017). The Use of Sugar Beet Pulp Lignin for the Production of Vanillin. Int. J. Biol. macromolecules 94, 345–354. 10.1016/j.ijbiomac.2016.10.004 27717789

[B2] Adair B. M. Cobb G. P. (1999). Improved Preparation of Small Biological Samples for Mercury Analysis Using Cold Vapor Atomic Absorption Spectroscopy. Chemosphere 38, 2951–2958. 10.1016/s0045-6535(98)00493-7 10214720

[B3] Ansari S. Bari A. Ullah R. Mathanmohun M. Veeraraghavan V. P. Sun Z. (2019). Gold Nanoparticles Synthesized with Smilax Glabra Rhizome Modulates the Anti-obesity Parameters in High-Fat Diet and Streptozotocin Induced Obese Diabetes Rat Model. J. Photochem. Photobiol. B: Biol. 201, 111643. 10.1016/j.jphotobiol.2019.111643 31698218

[B4] Arya S. S. Mahto B. K. Sengar M. S. Rookes J. E. Cahill D. M. Lenka S. K. (2022). Metabolic Engineering of Rice Cells with Vanillin Synthase Gene (VpVAN) to Produce Vanillin. Mol. Biotechnol., 1–12. 10.1007/s12033-022-00470-8 35192168

[B5] Arya S. S. Rookes J. E. Cahill D. M. Lenka S. K. (2021a). Vanillin: a Review on the Therapeutic Prospects of a Popular Flavouring Molecule. Adv. Tradit Med. (Adtm) 21, 1–17. 10.1007/s13596-020-00531-w

[B6] Arya S. S. Sharma M. M. Das R. K. Rookes J. Cahill D. Lenka S. K. (2019). Vanillin Mediated green Synthesis and Application of Gold Nanoparticles for Reversal of Antimicrobial Resistance in *Pseudomonas aeruginosa* Clinical Isolates. Heliyon 5, e02021. 10.1016/j.heliyon.2019.e02021 31312733 PMC6609825

[B7] Arya S. S. Sharma M. M. Rookes J. E. Cahill D. M. Lenka S. K. (2021b). Vanilla Modulates the Activity of Antibiotics and Inhibits Efflux Pumps in Drug-Resistant *Pseudomonas aeruginosa* . Biologia 76, 781–791. 10.2478/s11756-020-00617-5

[B8] Balalakshmi C. Gopinath K. Govindarajan M. Lokesh R. Arumugam A. Alharbi N. S. (2017). Green Synthesis of Gold Nanoparticles Using a Cheap *Sphaeranthus indicus* Extract: Impact on Plant Cells and the Aquatic Crustacean Artemia Nauplii. J. Photochem. Photobiol. B: Biol. 173, 598–605. 10.1016/j.jphotobiol.2017.06.040 28697477

[B9] Boldeiu A. Simion M. Mihalache I. Radoi A. Banu M. Varasteanu P. (2019). Comparative Analysis of Honey and Citrate Stabilized Gold Nanoparticles: *In Vitro* Interaction with Proteins and Toxicity Studies. J. Photochem. Photobiol. B: Biol. 197, 111519. 10.1016/j.jphotobiol.2019.111519 31228688

[B10] Bonciu E. Firbas P. Fontanetti C. S. Wusheng J. Karaismailoğlu M. C. Liu D. (2018). An Evaluation for the Standardization of theAllium Cepatest as Cytotoxicity and Genotoxicity Assay. Caryologia 71, 191–209. 10.1080/00087114.2018.1503496

[B11] Cao M. Li J. Tang J. Chen C. Zhao Y. (2016). Gold Nanomaterials in Consumer Cosmetics Nanoproducts: Analyses, Characterization, and Dermal Safety Assessment. Small 12, 5488–5496. 10.1002/smll.201601574 27562146

[B12] Cao Y. Xie Y. Liu L. Xiao A. Li Y. Zhang C. (2017). Influence of Phytochemicals on the Biocompatibility of Inorganic Nanoparticles: a State-Of-The-Art Review. Phytochem. Rev. 16, 555–563. 10.1007/s11101-017-9490-8

[B13] Charbgoo F. Nejabat M. Abnous K. Soltani F. Taghdisi S. M. Alibolandi M. (2018). Gold Nanoparticle Should Understand Protein corona for Being a Clinical Nanomaterial. J. controlled release 272, 39–53. 10.1016/j.jconrel.2018.01.002 29305922

[B14] Cheng X. Tian X. Wu A. Li J. Tian J. Chong Y. (2015). Protein corona Influences Cellular Uptake of Gold Nanoparticles by Phagocytic and Nonphagocytic Cells in a Size-dependent Manner. ACS Appl. Mater. Inter. 7, 20568–20575. 10.1021/acsami.5b04290 26364560

[B15] Cvjetko P. Milošić A. Domijan A.-M. Vinković Vrček I. Tolić S. Peharec Štefanić P. (2017). Toxicity of Silver Ions and Differently Coated Silver Nanoparticles in Allium cepa Roots. Ecotoxicology Environ. Saf. 137, 18–28. 10.1016/j.ecoenv.2016.11.009 27894021

[B16] Das S. Debnath N. Pradhan S. Goswami A. (2017). Enhancement of Photon Absorption in the Light-Harvesting Complex of Isolated Chloroplast in the Presence of Plasmonic Gold Nanosol-A Nanobionic Approach towards Photosynthesis and Plant Primary Growth Augmentation. Gold Bull. 50, 247–257. 10.1007/s13404-017-0214-z

[B17] Debnath P. Mondal A. Hajra A. Das C. Mondal N. K. (2018). Cytogenetic Effects of Silver and Gold Nanoparticles on Allium cepa Roots. J. Genet. Eng. Biotechnol. 16, 519–526. 10.1016/j.jgeb.2018.07.007 30733769 PMC6353767

[B18] Deng F. Wang S. Xin H. (2016). Toxicity of CuO Nanoparticles to Structure and Metabolic Activity of Allium cepa Root Tips. Bull. Environ. Contam. Toxicol. 97, 702–708. 10.1007/s00128-016-1934-0 27704188

[B19] Ding Y. Bai X. Ye Z. Gong D. Cao J. Hua Z. (2019). Humic Acid Regulation of the Environmental Behavior and Phytotoxicity of Silver Nanoparticles to Lemna Minor. Environ. Sci. Nano 6, 3712–3722. 10.1039/c9en00980a

[B20] Ekvall M. T. Hedberg J. Odnevall Wallinder I. Malmendal A. Hansson L. A. Cedervall T. (2021). Adsorption of Bio-Organic Eco-corona Molecules Reduces the Toxic Response to Metallic Nanoparticles in Daphnia magna. Sci. Rep. 11, 10784–10811. 10.1038/s41598-021-90053-5 34031463 PMC8144400

[B21] Fadoju O. M. Osinowo O. A. Ogunsuyi O. I. Oyeyemi I. T. Alabi O. A. Alimba C. G. (2020). Interaction of Titanium Dioxide and Zinc Oxide Nanoparticles Induced Cytogenotoxicity in Allium cepa. The Nucleus, 1–8. 10.1007/s13237-020-00308-1

[B22] Falahati M. Attar F. Sharifi M. Haertlé T. Berret J.-F. Khan R. H. (2019). A Health Concern Regarding the Protein corona, Aggregation and Disaggregation. Biochim. Biophys. Acta (Bba) - Gen. Subjects 1863, 971–991. 10.1016/j.bbagen.2019.02.012 PMC7115795 30802594

[B23] Fatoni A. Hariani P. L. Hermansyah H. Lesbani A. (2018). Synthesis and Characterization of Chitosan Linked by Methylene Bridge and Schiff Base of 4,4-Diaminodiphenyl Ether-Vanillin. Indones. J. Chem. 18, 92–101. 10.22146/ijc.25866

[B24] Fytianos G. Rahdar A. Kyzas G. Z. (2020). Nanomaterials in Cosmetics: Recent Updates. Nanomaterials 10, 979. 10.3390/nano10050979 PMC7279536 32443655

[B25] Gechev T. S. Van Breusegem F. Stone J. M. Denev I. Laloi C. (2006). Reactive Oxygen Species as Signals that Modulate Plant Stress Responses and Programmed Cell Death. Bioessays 28, 1091–1101. 10.1002/bies.20493 17041898

[B26] Gomez V. Irusta S. Balas F. Navascues N. Santamaria J. (2014). Unintended Emission of Nanoparticle Aerosols during Common Laboratory Handling Operations. J. Hazard. Mater. 279, 75–84. 10.1016/j.jhazmat.2014.06.064 25038576

[B27] Gopinath K. Venkatesh K. S. Ilangovan R. Sankaranarayanan K. Arumugam A. (2013). Green Synthesis of Gold Nanoparticles from Leaf Extract of Terminalia Arjuna, for the Enhanced Mitotic Cell Division and Pollen Germination Activity. Ind. crops Prod. 50, 737–742. 10.1016/j.indcrop.2013.08.060

[B28] Govindaraju S. Rengaraj A. Arivazhagan R. Huh Y.-S. Yun K. (2018). Curcumin-conjugated Gold Clusters for Bioimaging and Anticancer Applications. Bioconjug. Chem. 29, 363–370. 10.1021/acs.bioconjchem.7b00683 29323877

[B29] Grandviewresearch (2019). Gold Nanoparticles Market Worth $6.33. Billion By 2025. *Grand View Research* Accessed (15/03/2021), Available at: https://www.grandviewresearch.com/press-release/global-gold-nanoparticles-market .

[B30] Grant W. F. (1982). Chromosome Aberration Assays in Allium. Mutat. Research/Reviews Genet. Toxicol. 99, 273–291. 10.1016/0165-1110(82)90046-x 7177154

[B31] He X. Pan Y. Zhang J. Li Y. Ma Y. Zhang P. (2015). Quantifying the Total Ionic Release from Nanoparticles after Particle-Cell Contact. Environ. Pollut. 196, 194–200. 10.1016/j.envpol.2014.09.021 25463714

[B32] Janani B. Raju L. L. Thomas A. M. Alyemeni M. N. Dudin G. A. Wijaya L. (2021). Impact of Bovine Serum Albumin - A Protein corona on Toxicity of ZnO NPs in Environmental Model Systems of Plant, Bacteria, Algae and Crustaceans. Chemosphere 270, 128629. 10.1016/j.chemosphere.2020.128629 33168289

[B33] Katsnelson B. Privalova L. Gurvich V. Makeyev O. Shur V. Beikin Y. (2013). Comparative *In Vivo* Assessment of Some Adverse Bioeffects of Equidimensional Gold and Silver Nanoparticles and the Attenuation of Nanosilver's Effects with a Complex of Innocuous Bioprotectors. Ijms 14, 2449–2483. 10.3390/ijms14022449 23354478 PMC3587996

[B34] Kumar D. Rajeshwari A. Jadon P. S. Chaudhuri G. Mukherjee A. Chandrasekaran N. (2015). Cytogenetic Studies of Chromium (III) Oxide Nanoparticles on Allium cepa Root Tip Cells. J. Environ. Sci. 38, 150–157. 10.1016/j.jes.2015.03.038 26702979

[B35] Kumari M. Khan S. S. Pakrashi S. Mukherjee A. Chandrasekaran N. (2011). Cytogenetic and Genotoxic Effects of Zinc Oxide Nanoparticles on Root Cells of Allium cepa. J. Hazard. Mater. 190, 613–621. 10.1016/j.jhazmat.2011.03.095 21501923

[B36] Kumawat M. Madhyastha H. Umapathi A. Singh M. Revaprasadu N. Daima H. K. (2022). Surface Engineered Peroxidase-Mimicking Gold Nanoparticles to Subside Cell Inflammation. Langmuir 38, 1877–1887. 10.1021/acs.langmuir.1c03088 35099982

[B37] Kyriazi M.-E. Giust D. El-Sagheer A. H. Lackie P. M. Muskens O. L. Brown T. (2018). Multiplexed mRNA Sensing and Combinatorial-Targeted Drug Delivery Using DNA-Gold Nanoparticle Dimers. Acs Nano 12, 3333–3340. 10.1021/acsnano.7b08620 29557641

[B38] Li H. Ye X. Guo X. Geng Z. Wang G. (2016). Effects of Surface Ligands on the Uptake and Transport of Gold Nanoparticles in rice and Tomato. J. Hazard. Mater. 314, 188–196. 10.1016/j.jhazmat.2016.04.043 27131459

[B39] Li S. Wang S. Yan B. Yue T. (2021). Surface Properties of Nanoparticles Dictate Their Toxicity by Regulating Adsorption of Humic Acid Molecules. ACS Sustain. Chem. Eng. 9, 13705–13716. 10.1021/acssuschemeng.1c02795

[B40] Li Z. Xu Y. Fu J. Zhu H. Qian Y. (2018). Monitoring of Au(iii) Species in Plants Using a Selective Fluorescent Probe. Chem. Commun. 54, 888–891. 10.1039/c7cc08333e 29164199

[B41] Liman R. Başbuğ B. Ali M. M. Acikbas Y. Ciğerci İ. H. (2021). Cytotoxic and Genotoxic Assessment of Tungsten Oxide Nanoparticles in Allium cepa Cells by Allium Ana-Telophase and Comet Assays. J. Appl. Genet. 62, 85–92. 10.1007/s13353-020-00608-x 33409932

[B42] Liman R. (2013). Genotoxic Effects of Bismuth (III) Oxide Nanoparticles by Allium and Comet Assay. Chemosphere 93, 269–273. 10.1016/j.chemosphere.2013.04.076 23790828

[B43] Liu N. Tang M. Ding J. (2020). The Interaction between Nanoparticles-Protein corona Complex and Cells and its Toxic Effect on Cells. Chemosphere 245, 125624. 10.1016/j.chemosphere.2019.125624 31864050

[B44] Lopez-Chaves C. Soto-Alvaredo J. Montes-Bayon M. Bettmer J. Llopis J. Sanchez-Gonzalez C. (2018). Gold Nanoparticles: Distribution, Bioaccumulation and Toxicity. *In Vitro* and *In Vivo* Studies. Nanomedicine: Nanotechnology, Biol. Med. 14, 1–12. 10.1016/j.nano.2017.08.011 28882675

[B45] Lovecká P. Macůrková A. Záruba K. Hubáček T. Siegel J. Valentová O. (2021). Genomic Damage Induced in Nicotiana Tabacum L. Plants by Colloidal Solution with Silver and Gold Nanoparticles. Plants 10, 1260. 34205810 10.3390/plants10061260 PMC8234410

[B46] Maity S. Chatterjee A. Guchhait R. De S. Pramanick K. (2020). Cytogenotoxic Potential of a Hazardous Material, Polystyrene Microparticles on Allium cepa L. J. Hazard. Mater. 385, 121560. 10.1016/j.jhazmat.2019.121560 31732349

[B47] Mangalampalli B. Dumala N. Grover P. (2018). Allium cepa Root Tip Assay in Assessment of Toxicity of Magnesium Oxide Nanoparticles and Microparticles. J. Environ. Sci. 66, 125–137. 10.1016/j.jes.2017.05.012 29628079

[B48] Mosquera J. García I. Henriksen-Lacey M. Martínez-Calvo M. Dhanjani M. Mascareñas J. L. (2020). Reversible Control of Protein Corona Formation on Gold Nanoparticles Using Host-Guest Interactions. ACS nano 14, 5382–5391. 10.1021/acsnano.9b08752 32105057 PMC7254833

[B49] Mukherjee S. Nethi S. K. Patra C. R. (2017). “Green Synthesized Gold Nanoparticles for Future Biomedical Applications,” in Particulate Technology for Delivery of Therapeutics (Springer), 359–393. 10.1007/978-981-10-3647-7_11

[B50] Naraginti S. Kumari P. L. Das R. K. Sivakumar A. Patil S. H. Andhalkar V. V. (2016). Amelioration of Excision Wounds by Topical Application of green Synthesized, Formulated Silver and Gold Nanoparticles in Albino Wistar Rats. Mater. Sci. Eng. C 62, 293–300. 10.1016/j.msec.2016.01.069 26952426

[B51] Ovais M. Ahmad I. Khalil A. T. Mukherjee S. Javed R. Ayaz M. (2018). Wound Healing Applications of Biogenic Colloidal Silver and Gold Nanoparticles: Recent Trends and Future Prospects. Appl. Microbiol. Biotechnol. 102, 4305–4318. 10.1007/s00253-018-8939-z 29589095

[B52] Pesnya D. S. (2013). Cytogenetic Effects of Chitosan-Capped Silver Nanoparticles in theAllium Cepatest. Caryologia 66, 275–281. 10.1080/00087114.2013.852342

[B53] Piella J. Bastús N. G. Puntes V. (2017). Size-Dependent Protein-Nanoparticle Interactions in Citrate-Stabilized Gold Nanoparticles: The Emergence of the Protein Corona. Bioconjug. Chem. 28, 88–97. 10.1021/acs.bioconjchem.6b00575 27997136

[B54] Qin L. Zeng G. Lai C. Huang D. Xu P. Zhang C. (2018). "Gold rush" in Modern Science: Fabrication Strategies and Typical Advanced Applications of Gold Nanoparticles in Sensing. Coord. Chem. Rev. 359, 1–31. 10.1016/j.ccr.2018.01.006

[B55] Rajeshwari A. Kavitha S. Alex S. A. Kumar D. Mukherjee A. Chandrasekaran N. (2015). Cytotoxicity of Aluminum Oxide Nanoparticles on Allium cepa Root Tip-Effects of Oxidative Stress Generation and Biouptake. Environ. Sci. Pollut. Res. 22, 11057–11066. 10.1007/s11356-015-4355-4 25794585

[B56] Rajeshwari A. Roy B. Chandrasekaran N. Mukherjee A. (2016a). Cytogenetic Evaluation of Gold Nanorods Using Allium cepa Test. Plant Physiol. Biochem. 109, 209–219. 10.1016/j.plaphy.2016.10.003 27744263

[B57] Rajeshwari A. Suresh S. Chandrasekaran N. Mukherjee A. (2016b). Toxicity Evaluation of Gold Nanoparticles Using an Allium cepa Bioassay. RSC Adv. 6, 24000–24009. 10.1039/c6ra04712b PMC13353057 42433444

[B58] Sabo-Attwood T. Unrine J. M. Stone J. W. Murphy C. J. Ghoshroy S. Blom D. (2012). Uptake, Distribution and Toxicity of Gold Nanoparticles in Tobacco (Nicotiana Xanthi) Seedlings. Nanotoxicology 6, 353–360. 10.3109/17435390.2011.579631 21574812

[B59] Saha N. Dutta Gupta S. (2017). Low-dose Toxicity of Biogenic Silver Nanoparticles Fabricated by Swertia Chirata on Root Tips and Flower Buds of Allium cepa. J. Hazard. Mater. 330, 18–28. 10.1016/j.jhazmat.2017.01.021 28208089

[B60] Sani A. Cao C. Cui D. (2021). Toxicity of Gold Nanoparticles (AuNPs): A Review. Biochem. Biophys. Rep. 26, 100991. 10.1016/j.bbrep.2021.100991 33912692 PMC8063742

[B61] Scherer M. D. Sposito J. C. V. Falco W. F. Grisolia A. B. Andrade L. H. C. Lima S. M. (2019). Cytotoxic and Genotoxic Effects of Silver Nanoparticles on Meristematic Cells of Allium cepa Roots: A Close Analysis of Particle Size Dependence. Sci. Total Environ. 660, 459–467. 10.1016/j.scitotenv.2018.12.444 30640113

[B62] Siegel J. Záruba K. Švorčík V. Kroumanová K. Burketová L. Martinec J. (2018). Round-shape Gold Nanoparticles: Effect of Particle Size and Concentration on *Arabidopsis thaliana* Root Growth. Nanoscale Res. Lett. 13, 95. 10.1186/s11671-018-2510-9 29637317 PMC5893504

[B63] Smaka-Kincl V. Stegnar P. Lovka M. Toman M. J. (1996). The Evaluation of Waste, Surface and Ground Water Quality Using the Allium Test Procedure. Mutat. Research/Genetic Toxicol. 368, 171–179. 10.1016/s0165-1218(96)90059-2 8692223

[B64] Soltys D. Rudzińska-Langwald A. Kurek W. Gniazdowska A. Sliwinska E. Bogatek R. (2011). Cyanamide Mode of Action during Inhibition of Onion (Allium cepa L.) Root Growth Involves Disturbances in Cell Division and Cytoskeleton Formation. Planta 234, 609–621. 10.1007/s00425-011-1429-5 21573814 PMC3162148

[B65] Song C. Li X. Wang S. Meng Q. (2016). Enhanced Conversion and Stability of Biosynthetic Selenium Nanoparticles Using Fetal Bovine Serum. RSC Adv. 6, 103948–103954. 10.1039/c6ra22747c

[B66] Souza I. R. Silva L. R. Fernandes L. S. P. Salgado L. D. Silva de Assis H. C. Firak D. S. (2020). Visible-light Reduced Silver Nanoparticles' Toxicity in Allium cepa Test System. Environ. Pollut. 257, 113551. 10.1016/j.envpol.2019.113551 31801672

[B67] Sztandera K. Gorzkiewicz M. Klajnert-Maculewicz B. (2018). Gold Nanoparticles in Cancer Treatment. Mol. Pharmaceutics 16, 1–23. 10.1021/acs.molpharmaceut.8b00810 30452861

[B68] Taylor A. F. Rylott E. L. Anderson C. W. N. Bruce N. C. (2014). Investigating the Toxicity, Uptake, Nanoparticle Formation and Genetic Response of Plants to Gold. PLOS one 9, e93793. 10.1371/journal.pone.0093793 24736522 PMC3988041

[B69] Tedesco S. Laughinghouse Iv H. (2012). Bioindicator of Genotoxicity: The Allium cepa Test. Environmental contamination, 137–156.

[B70] Tefera W. Liu T. Lu L. Ge J. Webb S. M. Seifu W. (2020). Micro-XRF Mapping and Quantitative Assessment of Cd in rice (Oryza Sativa L.) Roots. Ecotoxicology Environ. Saf. 193, 110245. 10.1016/j.ecoenv.2020.110245 32092577

[B71] Tsi Ndeh N. Maensiri S. Maensiri D. (2017). The Effect of green Synthesized Gold Nanoparticles on rice Germination and Roots. Adv. Nat. Sci. Nanosci. Nanotechnol. 8, 035008. 10.1088/2043-6254/aa724a

[B72] Umapathi A. Pn N. Madhyastha H. Singh M. Madhyastha R. Maruyama M. (2020). Curcumin and Isonicotinic Acid Hydrazide Functionalized Gold Nanoparticles for Selective Anticancer Action. Colloids Surf. A: Physicochemical Eng. Aspects 607, 125484. 10.1016/j.colsurfa.2020.125484

[B73] Venzhik Y. V. Moshkov I. E. Dykman L. A. (2021). Gold Nanoparticles in Plant Physiology: Principal Effects and Prospects of Application. Russ. J. Plant Physiol. 68, 401–412. 10.1134/s1021443721020205

[B74] Wheeler K. E. Chetwynd A. J. Fahy K. M. Hong B. S. Tochihuitl J. A. Foster L. A. (2021). Environmental Dimensions of the Protein corona. Nat. Nanotechnol. 16, 617–629. 10.1038/s41565-021-00924-1 34117462

[B75] Who (1985). Guide to Short-Term Tests for Detecting Mutagenic and Carcinogenic Chemicals. Geneva, Switzerland: World Health Organization.

[B76] Ye X. Li H. Wang Q. Chai R. Ma C. Gao H. (2018). Influence of Aspartic Acid and Lysine on the Uptake of Gold Nanoparticles in rice. Ecotoxicology Environ. Saf. 148, 418–425. 10.1016/j.ecoenv.2017.10.056 29101886

[B79] Zhang H. Goh N. S. Wang J. W. Pinals R. L. González-Grandío E. Demirer G. S. (2022). Nanoparticle Cellular Internalization is Not Required for RNA Delivery to Mature Plant Leaves. Nat. Nanotechnol. 17 (2), 197–205. 34811553 10.1038/s41565-021-01018-8 PMC10519342

[B77] Zhu Z.-J. Wang H. Yan B. Zheng H. Jiang Y. Miranda O. R. (2012). Effect of Surface Charge on the Uptake and Distribution of Gold Nanoparticles in Four Plant Species. Environ. Sci. Technol. 46, 12391–12398. 10.1021/es301977w 23102049

[B78] Zong J. Cobb S. L. Cameron N. R. (2017). Peptide-functionalized Gold Nanoparticles: Versatile Biomaterials for Diagnostic and Therapeutic Applications. Biomater. Sci. 5, 872–886. 10.1039/c7bm00006e 28304023

