# Supplementary material for: Reduced Genotoxicity of Gold Nanoparticles With Protein Corona in Allium cepa
Source: Front Bioeng Biotechnol. 2022 Apr 5;10:849464. doi: 10.3389/fbioe.2022.849464 (PMC9016219; doi:10.3389/fbioe.2022.849464)
Supplement: Supplementary file 1 [file DataSheet1.docx]

Supplementary Material

**Reduced genotoxicity of gold nanoparticles with protein corona in *Allium cepa* plant**

Sagar S. Arya^1,2^, James E. Rookes^2^, David M. Cahill^2^, Sangram K. Lenka^1^*

*^1^The Energy and Resources Institute, TERI-Deakin Nano Biotechnology Centre, Gurugram, Haryana, 122001, India*

*^2^School of Life and Environmental Sciences, Deakin University, Waurn Ponds Campus, Geelong, VIC, 3216, Australia*

Correspondence: [keshari2u@gmail.com](mailto:keshari2u@gmail.com)


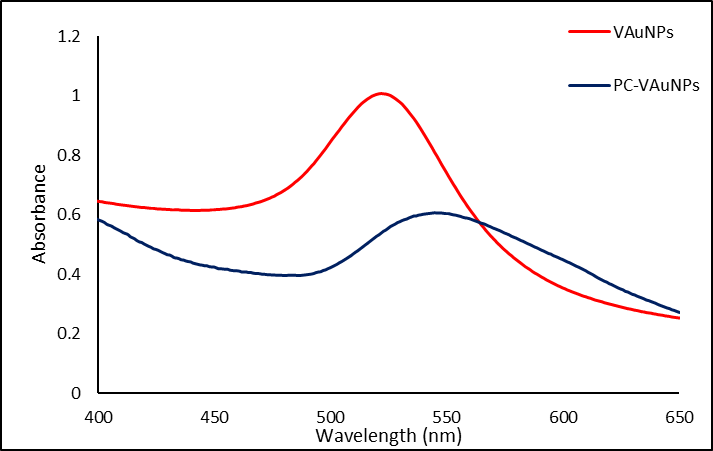


Figure S1: UV-visible spectrum of VAuNPs and PC-VAuNPs.

**Table 1S.** Elemental distribution in meristematic and elongation zones of *Allium cepa* roots. (MZ – meristematic zone; EZ – elongation zone; ‘-’ – not detected).

| Elements | Concentration (wt %) | | | | | | |
| --- | --- | --- | --- | --- | --- | --- | --- |
|  | **Control** | | **VAuNPs (200 µg/mL)** | | **PC-VAuNPs (200 µg/mL)** | |  |
|  | **MZ** | **EZ** | **MZ** | **EZ** | **MZ** | **EZ** |  |
| **Potassium (K)** | 84.47 | 84.81 | 80.20 | 87.41 | 79.68 | 85.81 |  |
| **Sulphur (S)** | 9.44 | 7.17 | 10.10 | 5.88 | 9.36 | 6.56 |  |
| **Phosphorous (P)** | 6.07 | 7.06 | - | - | 6.97 | 6.02 |  |
| **Lead (Pb)** | - | - | - | - | 2.09 | 1.00 |  |
| **Gold (Au)** | - | - | 7.03 | 5.42 | 1.85 | 0.58 |  |
| **Iron (Fe)** | - | - | 1.88 | - | - | - |  |
| **Zinc (Zn)** | - | 0.79 | - | 1.27 | - | - |  |
| **Chromium (Cr)** | - | - | 0.56 | - | - | - |  |
| **Gallium (Ga)** | - | - | 0.19 | - | - | - |  |
| **Cobalt (Co)** | - | 0.15 | - | - | - | - |  |
